# Superior canal dehiscence in a patient with three failed stapedectomy operations for otosclerosis: a case report

**DOI:** 10.1186/1752-1947-5-47

**Published:** 2011-02-03

**Authors:** Martin Lehmann, Jörg Ebmeyer, Tahwinder Upile, Holger H Sudhoff

**Affiliations:** 1 Department of Otolaryngology, Head and Neck Surgery, Bielefeld Academic Teaching Hospital, Münster University, Münster, Germany

## Abstract

**Introduction:**

This case illustrates that superior semicircular canal dehiscence syndrome can be associated with a "pseudo"-conductive hearing loss, a symptom that overlaps with the clinical appearance of otosclerosis.

**Case presentation:**

We present the case of a 48-year-old German Caucasian woman presenting with hearing loss on the left side and vertigo. She had undergone three previous stapedectomies for hearing improvement. Reformatted high-resolution computed tomographic scanning and the patient's history confirmed the diagnosis of concurrent canal dehiscence syndrome.

**Conclusion:**

Failure of hearing improvement after otosclerosis surgery may indicate an alternative underlying diagnosis which should be explored by further appropriate evaluation.

## Introduction

Superior semicircular canal dehiscence is an abnormal exposure of the vestibular membranous labyrinth in the middle cranial fossa. Superior semicircular canal dehiscence syndrome (SCD) occurs when a loss of the bone normally covering the superior semicircular canal in the middle cranial fossa produces one or more of the following symptoms: conductive hearing loss, acute pressure- and sound-evoked vestibular symptoms and chronic dysequilibrium [1]. The correlation between these symptoms and bony dehiscence of the superior semicircular canal in the floor of the middle cranial fossa was first recognized and described by Minor [2].

## Case presentation

**Figure 1:**
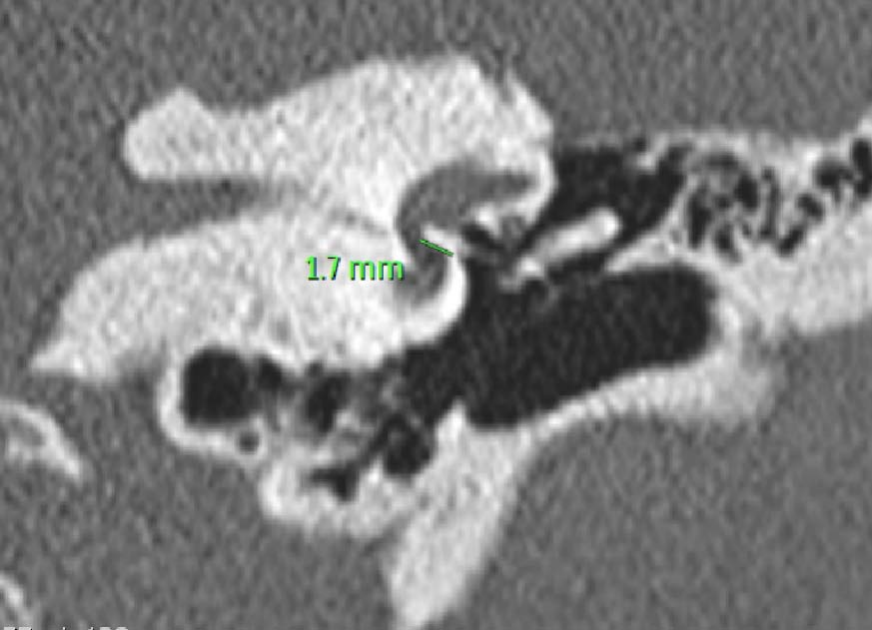
**High-resolution computed tomographic (CT) scan showing a left stapes prosthesis**. There appears to be an otospongiotic focus by the anterior lip of the stapes footplate. The platinum-Teflon prostheses appear to be extending deep into the vestibule.

We present the case of a 48-year-old German Caucasian woman who presented with hearing loss on the left side and vertigo. The patient had a history of three previous stapedectomy operations carried out elsewhere to improve her hearing loss (Figure 1). The first operation was performed for the diagnosis of otosclerosis. The next two operations were performed to improve her persistent hearing loss and vertigo.

**Figure 2:**
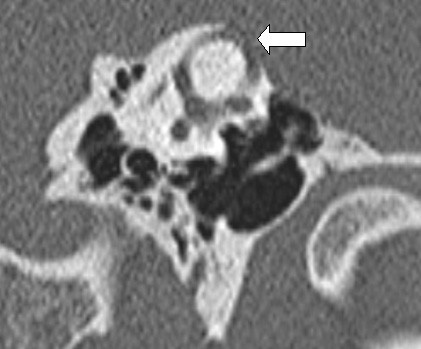
**High-resolution CT scan showing a left superior semicircular canal dehiscence (arrow)**.

After the third operation, the patient came to our unit with persistent amblyacousia as well as severe vertigo and headache. Pure tone audiometry showed a maximal conductive hearing loss. The patient located in her left ear the sound of a tuning fork pressed on the right ankle. This phenomenon suggested SCD. Further high-resolution computed tomographic (CT) scans and audiometery were performed. A CT scan revealed superior semicircular canal dehiscence (Figure 2).

## Discussion

We hypothesize that the otosclerotic focus in the oval window prevented the development of symptoms from this patient's SCD. Surgical stapedectomy created a third window and resulted in immediate postoperative imbalance and auditory symptoms.

SCD is one of the best documented and most investigated third-window lesions of the inner ear. We posit that patients with persistent audiovestibular symptoms after stapes surgery should be examined for the presence of SCD [3].

A combination of high-resolution CT scans and audiometry is recommended for diagnosis. The audiometric signs of SCD are conductive hearing loss with low-frequency bone conduction threshold better than 0dB(HL) and normal tympanometry with intact acoustic reflexes.

Auditory manifestations include hyperacusis to bone-conducted sounds and conductive hearing loss with normal acoustic reflexes. A directed patient history, documentation of upward and torsional nystagmus evoked by sound and/or pressure and radiologic findings are helpful in the diagnosis of SCD.

Acoustic reflexes and vestibular evoked myogenic potentials (VEMPs) aid in the identification of patients with an apparent conductive hearing loss with normal acoustic reflexes or those patients who are found to have an asymptomatic dehiscence on radiology [4]. The treatment involves avoidance of the precipitating stimuli [5].

The typical audiometric findings are of an air-bone gap in the low and middle frequencies (≤2,000 Hz) with no gap or only a small gap at higher frequencies. The low-frequency (<2,000 Hz) bone conduction thresholds are sometimes at supranormal levels, 0 to -20 dB or better. The lack of middle ear pathologic findings as a cause of the conductive hearing loss (CHL) in SCD has been well documented by a variety of diagnostic tests, such as tympanometry, acoustic reflexes, laser Doppler vibrometry, air-conducted VEMP testing, otoacoustic emission testing and exploration of the middle ear [6-13]. Definitive evidence that the SCD can cause CHL is demonstrated by resolution of the air-bone gap upon patching or plugging the dehiscence, as reported by various investigators [10,14]. The mechanism of CHL in a patient with SCD is a combination of an increase in air conduction thresholds combined with an improvement in bone conduction thresholds [15,16] as described above.

## Conclusion

In choosing treatment options, the severity of symptoms in each individual patient should be considered. Patients with minimal or minor symptoms should avoid provocative stimuli and undergo supportive measures such as vestibular rehabilitation or vestibular suppressants. These patients may require longer follow-up to ensure symptom resolution. Patients with disabling sound- or pressure-induced vertigo, imbalance or oscillopsia may require surgical treatment. The standard surgical options include middle fossa craniotomy for superior canal occlusion or resurfacing and transmastoid superior semicircular canal occlusion [6]. The aim of all of these surgical options is to occlude the superior semicircular canal to eliminate the third mobile inner ear window. The short- and long-term results depend on the approach and procedure. Another new surgical technique has been described recently by Silverstein and Van Ess [17], who occluded the round window niche using a transcanal approach and reported resolution or improvement of symptoms associated with SCD [17,18].

## Competing interests

The authors declare that they have no competing interests.

## Consent

Written informed consent was obtained from the patient for publication of this case report and accompanying images. A copy of the written consent is available for review by the Editor-in-Chief of this journal.

## Authors' contributions

ML and JE analyzed and interpreted the patient data regarding the otorhinolaryngological disease. TU and HS were major contributors in writing the manuscript. All authors read and approved the final manuscript.
